# Elevated Glutamate and Glutamine Levels in the Cerebrospinal Fluid of Patients With Probable Alzheimer's Disease and Depression

**DOI:** 10.3389/fpsyt.2018.00561

**Published:** 2018-11-06

**Authors:** Caroline Madeira, Charles Vargas-Lopes, Carlos Otávio Brandão, Taylor Reis, Jerson Laks, Rogerio Panizzutti, Sergio T. Ferreira

**Affiliations:** ^1^Institute of Biomedical Sciences, Federal University of Rio de Janeiro, Rio de Janeiro, Brazil; ^2^Institute of Psychiatry, Federal University of Rio de Janeiro, Rio de Janeiro, Brazil; ^3^Institute of Medical Biochemistry Leopoldo de Meis, Federal University of Rio de Janeiro, Rio de Janeiro, Brazil; ^4^Institute of Biophysics Carlos Chagas Filho, Federal University of Rio de Janeiro, Rio de Janeiro, Brazil

**Keywords:** Alzheimer's disease, depression, glutamate, glutamine, cerebrospinal fluid, innotest amyloid tau index

## Abstract

Recent evidence suggests that Alzheimer's disease (AD) and depression share common mechanisms of pathogenesis. In particular, deregulation of glutamate-mediated excitatory signaling may play a role in brain dysfunction in both AD and depression. We have investigated levels of glutamate and its precursor glutamine in the cerebrospinal fluid (CSF) of patients with a diagnosis of probable AD or major depression compared to healthy controls and patients with hydrocephalus. Patients with probable AD or major depression showed significantly increased CSF levels of glutamate and glutamine compared to healthy controls or hydrocephalus patients. Furthermore, CSF glutamate and glutamine levels were inversely correlated to the amyloid tau index, a biomarker for AD. Results suggest that glutamate and glutamine should be further explored as potential CSF biomarkers for AD and depression.

## Introduction

Epidemiological and clinical studies suggest an association between Alzheimer's disease (AD) and depression (1, 2), the latter being a risk factor for development of AD and other forms of dementia (3–5). About half of the patients with major depression show cognitive impairment that can be persistent and last even after remission of the acute phase of symptoms (6). This could mean cognitive impairment precedes or predisposes to depression or, alternatively, that depression produces persistent cognitive deficits (6, 7). Recent studies have further suggested that similar pathogenic mechanisms may underlie cognitive changes in depression and in AD. Indeed, we have demonstrated that soluble oligomers of the amyloid-β peptide (AβOs), neurotoxins that accumulate in the AD brain and are thought to cause synapse failure and memory loss in AD (8, 9), induce depressive-like behavior in mice (10, 11), providing insight into molecular/cellular mechanisms potentially connecting both disorders.

Glutamate is the major excitatory neurotransmitter in the mammalian brain. However, glutamate at high concentrations in the synaptic cleft is toxic and may result in neuronal death, a phenomenon generally termed excitotoxicity (12). Excitotoxicity mediated by aberrant activation of glutamate receptors, notably of N-methyl-D-aspartate (NMDA) receptors, has been related to the neuropathology of AD (13–16). Consistent with a role of abnormal NMDA receptor function in AD, memantine, an NMDA receptor blocker, is one of the few drugs in clinical use for treatment of moderate to severe AD (17, 18). Interestingly, NMDA receptor antagonists, in particular ketamine, also produce rapid and consistent antidepressant action in subjects with major depression (19, 20).

To prevent excitotoxicity after physiological neurotransmission, glutamate is rapidly removed from the synaptic cleft and converted into glutamine by glutamine synthetase in glial cells (21). Glutamine is then transported back to the presynaptic neuron, where it is converted to glutamate by glutaminase (22). Dysfunction in the glutamate-glutamine cycle could contribute to excitotoxicity mediated by glutamate (22).

Changes in glutamate and glutamine may occur in AD and depression, previous studies of glutamate and glutamine levels in the cerebrospinal fluid (CSF) present controversial findings. Some studies have reported that CSF glutamate levels in AD patients were higher than in the controls (23, 24) or MCI (25), whereas others studies have found decreased (26, 27) or unchanged glutamate levels in AD patients compared to healthy controls (28, 29). Studies that measured CSF glutamine levels have also reported controversial results, with studies reporting increased (30), decreased (28, 31) or no change in glutamine levels in AD patients compared with controls (23, 24, 26).

Controversial findings have also been observed in CSF glutamate and glutamine levels of patients with depression. One study found an increase in CSF glutamine levels (32), whereas another study showed a decrease in CSF glutamate levels in patients with depression compared to controls (33). Moreover, recent studies reported no differences in CSF glutamate and glutamine levels in patients with depression compared to controls (34, 35).

Here, we investigated whether changes in brain levels of glutamate and glutamine are present in major depression and AD. We have studied glutamate and glutamine levels in the CSF of age-matched patients with probable AD or major depression compared to two control groups: healthy subjects and patients with an unrelated neurological condition (normal pressure hydrocephalus). We further investigated the correlation between glutamate and glutamine levels, Mini-Mental State Examination (MMSE) scores, and the Innotest amyloid tau index, a biomarker for AD (36, 37).

## Materials and methods

### Human subjects

The current study was approved by the Committee of Research Ethics involving human subjects of the Institute of Psychiatry of Federal University of Rio de Janeiro (protocol # 32liv2/07). Subjects provided written informed consent before study admission.

All subjects underwent an evaluation comprising full medical history, physical and neurological examination, laboratory tests and neuropsychological assessments. The complete work up is detailed elsewhere (38). The severity of dementia was classified according to the Clinical Dementia Rating (CDR) (39). Mini-Mental State Examination (MMSE) (40) was additionally used to evaluate cognitive state. Exclusion criteria included more than 10 packs/year of cigarette smoking, alcohol abuse or other current or previous psychiatric or clinical disorder. The probable AD group included 21 subjects recruited from the AD Center of the Institute of Psychiatry of the Federal University of Rio de Janeiro. Subjects were diagnosed according to National Institute of Neurological and Communicative Disorders and Stroke (NINCDS), Alzheimer's Disease and Related Disorders Association (ADRDA) and Diagnostic and Statistical Manual of Mental Disorders (DSM-IV) criteria (41). The depression group included 9 subjects with major depression, recruited from the Institute of Psychiatry of the Federal University of Rio de Janeiro and diagnosed according to DSM-IV criteria. The severity of depression was measured by the Brazilian version of the Hamilton Depression Scale (HAM-D) (42–44). Only patients in the first episode of major depression were included in the study. The healthy control group included 10 subjects without any clinical disease or neuropsychiatric disorder. We also studied an additional control group including 9 patients with hydrocephalus, diagnosed according to International Classification of Diseases (ICD-10) (45). Both healthy and hydrocephalus subjects were recruited at Neurolife Laboratory, a private clinic specialized in CSF analysis in the city of Rio de Janeiro. Selected characteristics of each studied group are presented in Table 1. Detailed demographics of individual subjects are presented in Table S1.

**Table 1:** Characteristics of study subjects.

	**Control**	**AD**	**Major depression**	**Hydrocephalus**	**Statistics**
Age, years	70.7 (6.3)	72.1 (8.4)	69.8 (5.8)	74.6 (7.4)	0.71 (0.55)
Sex, male/female	3/7	9/12	0/9	5/4	5.90 (0.01)^*^
Education, years	7.9 (5.1)	4.8 (4.8)	2.7 (2.6)	7.6 (5.7)	2.66 (0.06)
Disease duration, months	N.A.	44.8 (28.2)	N.A.	24.7 (13.6)	N.A.
MMSE	27.1 (1.3)	12.7 (6.2)^a^	24.4 (2.2)	27.2 (1.8)	39.66 (0.0001)^*^
IATI	1.95 (0.40)	0.74 (0.34)^b^	1.58 (0.62)	1.67 (0.56)	18.29 (0.0001)^*^
HAM-D	N.A.	N.A.	15.2 (2.0)	N.A.	N.A.
Glutamate, μmol/l	6.16 (3.19)	17.28 (1.99)^c^	16.31 (3.81)^d^	9.05 (2.06)	50.83 (0.0001)^*^
Glutamine, μmol/l	359.3 (102.1)	534.0 (146.8)^e^	493.7 (151.8)	359.6 (108.2)	5.93 (0.002)^*^
Glutamate/glutamine ratio	0.0158 (0.0060)	0.0334 (0.0079)^f^	0.0325 (0.0082)^g^	0.0260 (0.0043)^h^	15.34 (0.0001)^*^

*Values are presented as means (standard deviation). Statistical significance of differences between groups was assessed by one-way ANOVA, F (p-value), followed by Bonferroni adjustment for multiple comparisons, except for sex distribution, which was assessed by the Chi-Square Test, X*^2^ *(p-value). Asterisks indicate statistically significant differences*.

*AD, Alzheimer's disease; MMSE, Mini Mental State Examination; IATI, Innotest amyloid tau index; HAM-D, Hamilton Depression Scale; N.A., Not applicable or not available*.

a *AD significantly different from control, hydrocephalus and depression (P* = *0.0001)*.

b *AD significantly different from control, hydrocephalus and depression (P = 0.0001)*.

c *AD significantly different from control and hydrocephalus (P = 0.0001)*.

d *Depression significantly different from control and hydrocephalus (P* = *0.0001)*.

e *AD significantly different from control and hydrocephalus (P* = *0.008 and P* = *0.012, respectively)*.

f *AD significantly different from control (P* = *0.0001)*.

g *Depression significantly different from control (P* = *0.0001)*.

h Hydrocephalus significantly different from control (P = 0.018).

Psychotropic medications used by probable AD patients were: rivastigmine (47.6%; *n* = 10), risperidone (38.1%; *n* = 8), memantine (28.6%; *n* = 6), donepezil (23.8%; *n* = 5), clonazepam (19.0%; *n* = 4), citalopram (4.8%; *n* = 1), trazodone (4.8%; *n* = 1), biperiden (4.8%; *n* = 1), escitalopram (4.8%; *n* = 1), and mirtazapine (4.8%; *n* = 1). Two (9.5%) patients with probable AD were not taking any medication at the time of the study. Medications used by major depression patients were: citalopram (11.1%; *n* = 1), clonazepam (33.3%; *n* = 3), fluoxetine (22.2%; *n* = 2), desvenlafaxine (11.1%; *n* = 1), paroxetine (11.1%; *n* = 1), buspirone (11.1%; *n* = 1), sertraline (11.1%; *n* = 1), venlafaxine (11.1%; *n* = 1). One (11.1%) patient with major depression was not taking any medication at the time of the study.

### CSF collection

CSF samples were collected by lumbar puncture in the L3-4 or L4-5 interspace at Neurolife Laboratories (Rio de Janeiro, Brazil) and immediately stored at −80°C. All lumbar punctures were performed between 10 am and noon to limit potential circadian fluctuation in CSF content.

### Glutamate and glutamine measurements in CSF

Glutamate and glutamine levels in CSF were measured by high performance liquid chromatography (HPLC) as previously described (46–48).

### Determination of the amyloid tau index

CSF concentrations of Aβ1-42, total tau (T-tau) and phosphorylated tau (P-tau181) were measured using commercially available enzyme-linked immunosorbent assays (ELISA INNOTEST p-tau-181, INNOTEST htau, INNOTEST β-amyloid (1-42) kits; Innogenetics, Gent, Belgium) according to manufacturer's instructions. The Innotest amyloid tau index (IATI) is a score that combines CSF levels of Aβ1-42 and T-tau (36, 37) and was calculated as:

IATI = Aβ_1−42_/(240+1.18^*^T-tau).

### Statistical analysis

Results are presented as means (±S.D.) unless otherwise indicated. Statistical significances between groups were determined by one-way analysis of variance (ANOVA) followed by Bonferroni test for multiple comparisons. Sex distribution was studied using Chi-Square. Interactions between years of education, age, disease duration and levels of glutamate or glutamine were studied using correlation analysis and were demonstrated using Pearson's correlation coefficient (*r*).

## Results

Patient groups did not differ in age, but the sex distribution was significantly different between groups (Table 1). However, logistic regression analysis did not reveal significant interactions between sex and levels of glutamate or glutamine (χ^2^ = 0.21, *p* = 0.64 for glutamate; χ^2^ = 0.93, *p* = 0.33 for glutamine). Groups also differed in terms of numbers of years of education (Table 1). However, no significant correlations were found between years of education and levels of glutamate or glutamine (*r* = −0.19, *p* = 0.18 for glutamate; *r* = −0.02, *p* = 0.87 for glutamine). Moreover, glutamate and glutamine levels were not significantly correlated to age (*r* = 0.04, *p* = 0.78 for glutamate; *r* = 0.22, *p* = 0.12 for glutamine) or disease duration (*r* = 0.22, *p* = 0.23 for glutamate; *r* = 0.03, *p* = 0.86 for glutamine). None of the medications in use by the patients showed a significant effect on glutamate and glutamine levels (Table S2).

**Figure 1:**
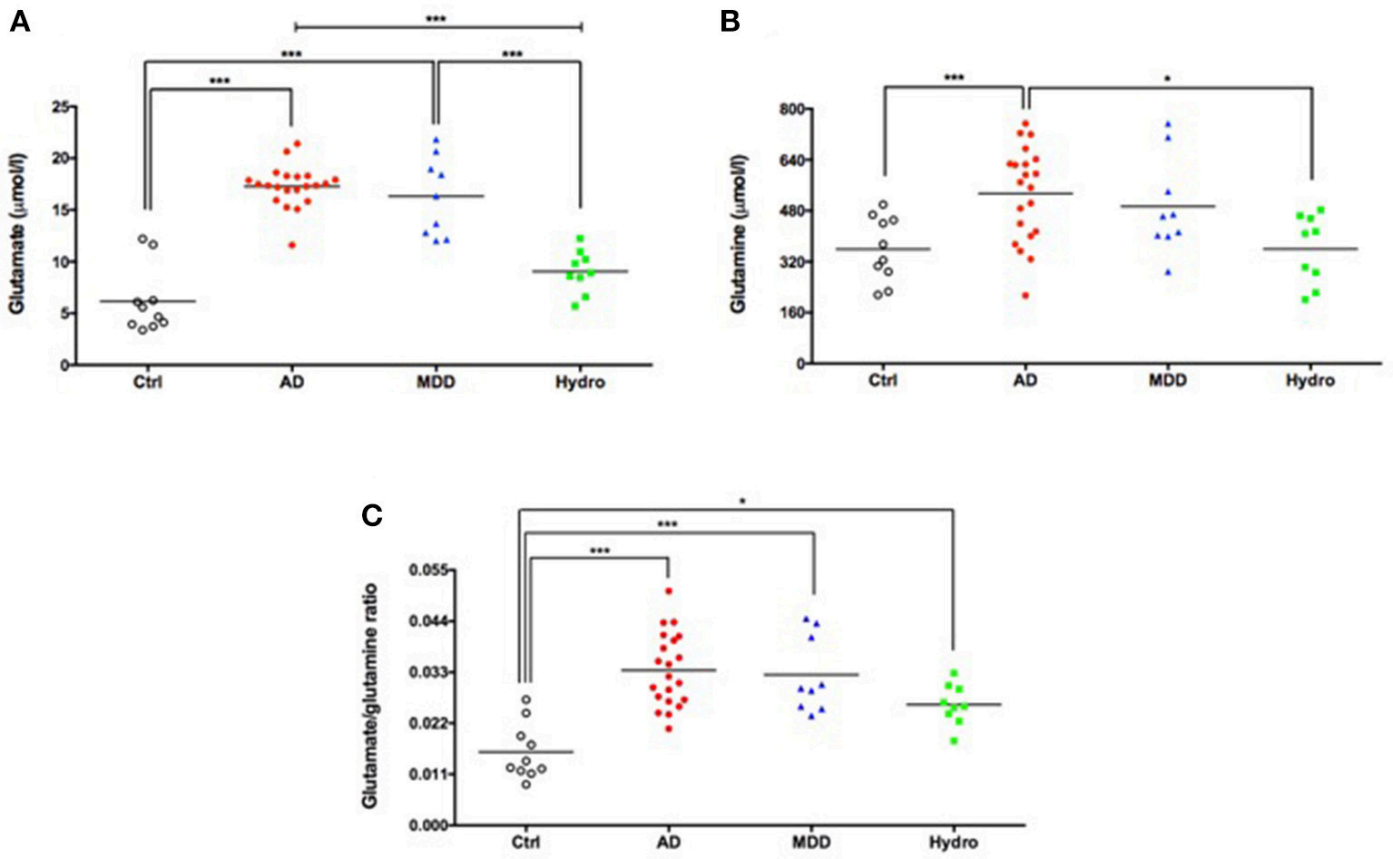
Increased CSF levels of glutamate **(A)**, glutamine **(B)** and glutamate/glutamine ratio **(C)** in patients with probable AD and major depression. Symbols correspond to individual subjects. Horizontal lines represent mean values for each group. Statistical significances assessed by one-way ANOVA followed by Bonferroni adjustment for multiple comparisons. ^*^P < 0.05; ^***^P < 0.001. AD, probable Alzheimer's disease; Ctrl, healthy controls; MDD, major depressive disorder; Hydro, hydrocephalus.

Mean CSF glutamate levels were significantly higher in patients with probable AD compared to healthy controls and hydrocephalus patients (*F* = 50.8, *p* < 0.0001) (Table 1; Figure 1A). Interestingly, CSF glutamate levels in the major depression group were similar to the levels found in the probable AD group (Table 1, Figure 1A). The sensitivity and specificity were 95.2 and 100%, respectively (using a cutoff of 13.63 μmol glutamate/l) for the diagnosis of probable AD compared to healthy controls (AUC = 0.99, *p* < 0.0001). The corresponding ROC curve is presented in Figure S1.

Mean glutamine levels were significantly higher in patients with probable AD than in healthy controls and in the hydrocephalus group (*F* = 5.92, *p* = 0.002). Moreover, the mean CSF glutamine level in the major depression group was similar to the mean level found in the group of patients with probable AD (Table 1; Figure 1B).

The glutamate/glutamine ratio, an index of glutamine-glutamate cycle in the brain (49), was significantly higher in all three patient groups (probable AD, major depression and hydrocephalus) compared to healthy controls (Table 1; Figure 1C). Thus, while measurements of CSF glutamate alone robustly separated probable AD and depressive patients from controls and hydrocephalus patients, the glutamate/glutamine ratio was elevated in all three disorders compared to controls, suggesting it was a less specific biomarker for AD and depression.

**Figure 2:**
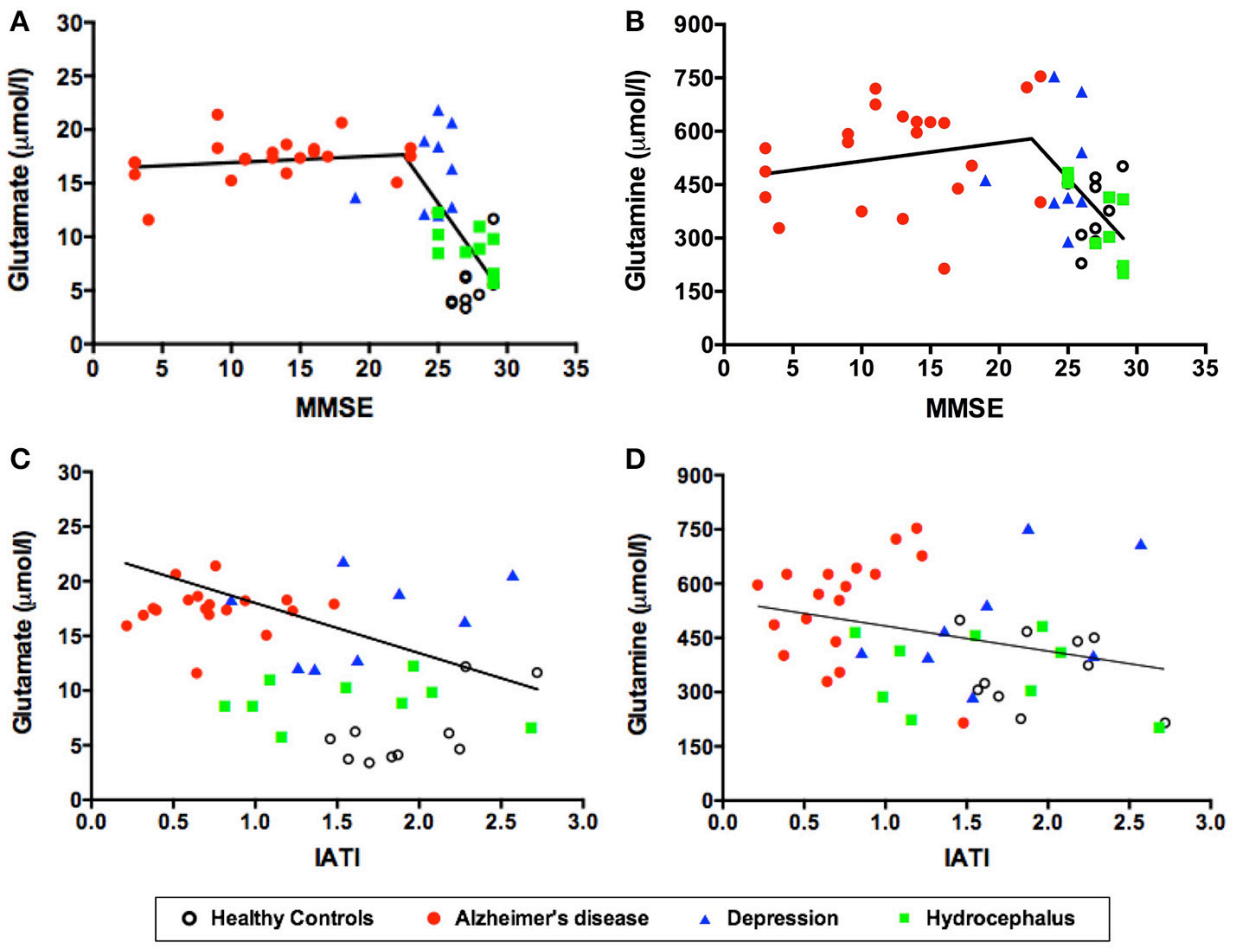
CSF levels of glutamate **(A)** and glutamine **(B)** as a function of MMSE scores. CSF levels of glutamate **(C)** and glutamine **(D)** as a function of the IATI index. Symbols correspond to individual subjects. Statistical significances assessed by Pearson correlation. MMSE, Mini Mental State Examination; IATI, INNOTEST amyloid/tau index.

As expected, the mean MMSE score was significantly lower in patients with probable AD than in healthy controls, major depression and hydrocephalus (Table 1). Interestingly, in subjects without dementia (MMSE above 23) (50), lower MMSE scores were significantly associated with higher CSF glutamate (*r* = −0.51, *p* = 0.006) (Figure 2A) and glutamine levels (*r* = −0.47, *p* = 0.013) (Figure 2B).

As also expected, the mean CSF Innotest amyloid tau index (IATI) was significantly lower in the AD group compared to the other three groups (Table 1). Remarkably, CSF glutamate levels were significantly and inversely correlated to the IATI across the four subject groups (*r* = −0.45, *p* = 0.002) (Figure 2C). Glutamine levels were also inversely correlated to the IATI, although the correlation was less robust and significant (*r* = −0.31, *p* = 0.04) (Figure 2D).

**Figure 3:**
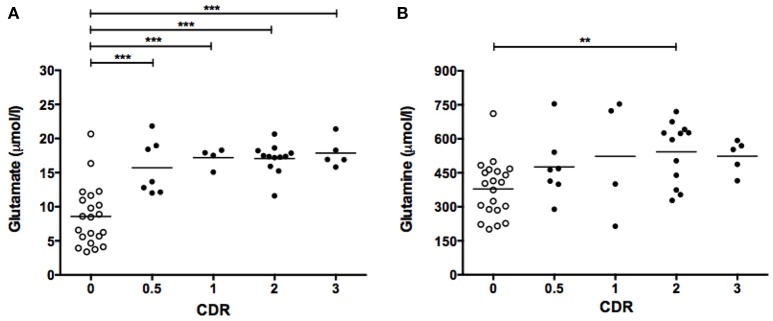
CSF levels of glutamate **(A)** and glutamine **(B)** as a function of CDR score. Symbols correspond to individual subjects. Horizontal lines represent mean values for each group. Statistical significances assessed by one-way ANOVA followed by Bonferroni adjustment for selected groups: CDR 0.5, 1, 2, and 3 vs. CDR 0. **P < 0.01; ***P < 0.001. CDR, Clinical Dementia Ratio.

Finally, CSF glutamate and glutamine levels were analyzed in subject groups separated by their clinical dementia rating (CDR) scores. Individuals with CDR 0.5, 1, 2, and 3 showed significantly elevated mean glutamate levels compared to non-cognitively impaired individuals (CDR 0) (Figure 3A). On the other hand, only the group with CDR 2 exhibited significantly higher glutamine than the CDR 0 group (Figure 3B).

## Discussion

We report increased CSF levels of glutamate and glutamine in AD and major depression compared to healthy controls and to patients with normal pressure hydrocephalus. Significantly, lower MMSE and IATI scores were correlated with higher glutamate and glutamine levels in our study cohort.

The current findings are consistent with previous studies reporting increased glutamate in the CSF of patients with probable AD (23, 24). In addition, CSF glutamate levels were found to be significantly elevated in patients with AD in comparison to patients with mild cognitive impairment (25). However, other groups have reported decreased (26, 27) or unchanged glutamate levels in patients with probable AD compared to healthy controls (28, 29). Moreover, our finding of increased CSF glutamine in AD patients is in accord with a previous study (30) but differs from other studies that reported a decrease (28, 31) or no change in glutamine levels in patients with probable AD compared to healthy controls (23, 24, 26). It is noteworthy that the criteria for diagnosis of probable AD in early studies included only clinical features (51), which could lead to greater heterogeneity amongst individuals included in the studies and, hence, to greater variability in results from CSF analysis. More recent revisions in diagnostic and research criteria have advocated the use of biomarkers as closely as possible to the pathological criteria for AD so as to increase the level of certainty in the diagnosis for research purposes (52, 53). All subjects with probable AD studied here were positive for the biomarker amyloid tau index (IATI), thus showing clear evidence of both amyloid-β and tau neuropathology. Interestingly, glutamate and glutamine levels were inversely correlated with individual IATI values across all groups of subjects. We further note that we have previously reported measurements of CSF levels of D-serine, L-serine and glycine in the same patient cohort investigated in the current study (48). We found that D-serine levels in patients with probable AD were significantly higher than in healthy controls, but there were no differences in L-serine and glycine levels.

We further found elevated CSF glutamate and glutamine in major depression patients, similar to levels found in the AD group and significantly higher than the levels found in both healthy control individuals and hydrocephalus patients. Previous studies have measured glutamate and glutamine in the CSF of patients with major depression, and they report controversial findings. Levine et al. (32) reported increased glutamine levels in the CSF of depressed patients compared to controls, whereas Frye et al. (33) showed decreased glutamate levels in depression. More recent studies reported no differences in glutamate or glutamine levels in the CSF of depressed patients compared to controls (34, 35). Of note, Levine et al. (32), Frye et al. (33) and Garakani et al. (34) evaluated middle-aged adult patients, while here we studied older patients. On the other hand, Hashimoto et al. (35) also studied older patients, but the MMSE scores average of their study was slightly higher than in our study. More studies are thus warranted to clarify whether glutamate and glutamine are altered in individuals affected by depression and belonging to different age groups. Increased CSF glutamate may be related to the neurodegenerative process thought to occur in major depression. Evidence indicates that inflammation leading to neurodegeneration plays an important role in depression (54–56). Glutamate excitotoxicity may be involved in this cascade as inflammatory mediators increase glutamate release and decrease glutamate uptake in the central nervous system (57–59).

CSF glutamate and glutamine levels were similar in age-matched patients with probable AD or depression, suggesting another shared mechanism of pathogenesis in these two disorders. Accordingly, a prospective study in a large cohort suggested that depression in the older adult is a prodrome rather than a risk factor for AD (60). Given the central role of glutamatergic neurotransmission in synaptic plasticity, learning and memory (61), as well as in regulation of mood (62), the impact of altered glutamate and glutamine levels on neuropathological mechanisms connecting dementia and depression warrants further investigation. Notably, evidence from animals models indicates that AD and depression involves shared mechanisms, such as hyperactivation of NMDA receptors through increased D-serine (48), inflammatory process (10, 11) and serotonergic signaling (11).

What is the significance of increased glutamate and glutamine levels in the CSF of AD patients? The increase in CSF glutamine may be due to increased activity of aspartate aminotransferase, an enzyme that forms glutamine, in the AD brain (30). A possible mechanism to explain the increase in CSF glutamate involves the build-up of Aβ oligomers (AβOs) in the AD brain. AβOs are increasingly recognized as proximal neurotoxins in AD (8, 9, 63, 64) and oligomer levels increase in AD brains. Of note, we have previously demonstrated that AβOs cause an increase in extracellular levels of glutamate in hippocampal neurons (65), and intracerebroventricular injection of AβOs induces both depressive-like behavior and cognitive deficits in mice (10, 11, 66, 67). It may thus be that AβO-instigated increases in brain glutamate levels underlie, at least in part, cognitive and mood alterations in AD.

Interestingly, we found that glutamate and (albeit somewhat less strongly) glutamine levels are significantly and inversely associated with the MMSE score in subjects without dementia (with MMSE above 23). This implies that alterations in CSF glutamate levels sensitively correlate with sub-clinical deterioration in cognitive performance. Thus, CSF measurements of glutamate/glutamine may serve as a biomarker of subtle cognitive changes that, while still within the range of normality, may reveal underlying mechanisms of pathogenesis potentially leading to future dementia. This could be of major clinical utility in terms of detecting pre-clinical dementia, with clear implications for inclusion of subjects in clinical trials and initiation of preventive/treatment strategies prior to overt cognitive deterioration.

A limitation of the present study was the absence of males in the group of patients with major depression. We note, however, that no sex differences in glutamate and glutamine levels were found in the other three patient groups studied. Additionally, the groups of patients with probable AD and major depression showed a clear trend (albeit not statistically significant) toward lower education than the control and hydrocephalus groups. However, analysis of differences in glutamate levels using education as a covariate confirmed that differences between patient groups remained significant (data not shown). Moreover, the cohort we have studied was of modest size, and it may not be representative of the population as a whole. However, the highly significant differences we have found between groups strongly suggest that the differences may hold in larger patient groups. Nevertheless, despite the current statistically robust results, we acknowledge that this is an initial investigation and, thus, a larger study is warranted to confirm and extend the validity of our findings.

In conclusion, glutamate and glutamine levels are increased in the CSF of patients with probable AD. Significantly increased glutamate levels were detected in CDR 0.5 patients, raising the possibility that determination of CSF glutamate levels might constitute an additional biomarker for pre-clinical cognitive deterioration or early stages of dementia. Finally, our finding that glutamate and glutamine levels were also increased in the CSF of older patients with major depression suggests that these amino acids may be involved in shared mechanisms of pathogenesis between AD and major depression.

## Author contributions

All authors certify that they have participated sufficiently in the work to take public responsibility for the content. RP and SF participated in the conception and design of study. CM, RP, and SF wrote the manuscript. CM and CV-L performed the analysis and interpretation of the data. CB, TR, and JL conduced analysis of patients.

### Conflict of interest statement

The authors declare that the research was conducted in the absence of any commercial or financial relationships that could be construed as a potential conflict of interest.

## Abbreviations

AD: Alzheimer's disease
ADRDA: Alzheimer's Disease and Related Disorders Association
ANOVA: Analysis of variance
Aβ: amyloid-β peptide
AβO: amyloid-β oligomer
CDR: Clinical Dementia Rating
CSF: cerebrospinal fluid
DSM-IV: Diagnostic and Statistical Manual of Mental Disorders
GLX: glutamate plus glutamine concentrations
HPLC: high performance liquid chromatography
IATI: Innotest amyloid tau index
ICD-10: International Classification of Diseases
MMSE: Mini-Mental State Examination
MRS: magnetic resonance spectroscopy
NINCDS: National Institute of Neurological and Communicative Disorders and Stroke
NMDA: N-methyl-D-aspartate
P-tau181: phosphorylated tau
T-tau: total tau.
