# Supplementary material for: Elevated Glutamate and Glutamine Levels in the Cerebrospinal Fluid of Patients With Probable Alzheimer's Disease and Depression
Source: Front Psychiatry. 2018 Nov 6;9:561. doi: 10.3389/fpsyt.2018.00561 (PMC6232456; doi:10.3389/fpsyt.2018.00561)
Supplement: Supplementary file 1 [file Data_Sheet_1.PDF]

Elevated glutamate and glutamine levels in the cerebrospinal fluid of patients with probable Alzheimer's disease and depression

Caroline Madeira[1], Charles Vargas-Lopes[1], Carlos Otávio Brandão[2], Taylor Reis[2], Jerson Laks[2], Rogerio Panizzutti[1,2,]*, Sergio T. Ferreira[3,4]*.

[1]Institute of Biomedical Sciences, [2]Institute of Psychiatry, [3]Institute of Medical Biochemistry Leopoldo de Meis, [4]Institute of Biophysics Carlos Chagas Filho, Federal University of Rio de Janeiro, Rio de Janeiro, RJ, Brazil.

*Correspondence should be addressed to:
Dr. Sergio T. Ferreira
ferreira@bioqmed.ufrj.br
or
Dr. Rogerio Panizzutti
rogerio@icb.ufrj.br

Supplemental Information

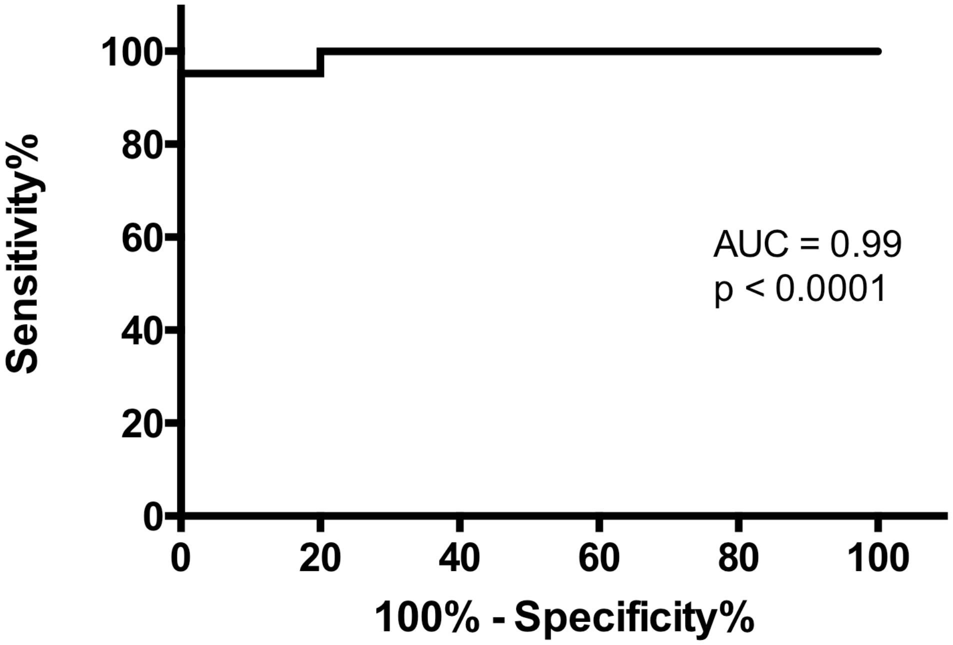

Figure S1 – Receiver-operating characteristics curve (ROC curve) of CSF glutamate levels for the diagnosis of probable AD compared to healthy controls.

Table S1 - Characteristics of each individual in CSF analysis.

Patient N°/Age (years)/Sex	CDR	Disease duration (years)	Education (years)	MMSE	HAM-D	Currently Medicated	Clinical diagnosis
C1/62/F	0	N.A.	16	27	N.A.	N.A.	Control
C2/77/M	0	N.A.	6	27	N.A.	N.A.	Control
C3/65/M	0	N.A.	9	29	N.A.	N.A.	Control
C4/76/M	0	N.A.	2	26	N.A.	N.A.	Control
C5/67/F	0	N.A.	16	28	N.A.	N.A.	Control
C6/73/F	0	N.A.	11	27	N.A.	N.A.	Control
C7/71/F	0	N.A.	6	26	N.A.	N.A.	Control
C8/72/F	0	N.A.	4	27	N.A.	N.A.	Control
C9/81/F	0	N.A.	7	25	N.A.	N.A.	Control
C10/63/F	0	N.A.	2	29	N.A.	N.A.	Control
A1/64/F	2	4.2	0	4	N.A.	C	AD
A2/78/M	2	8.2	4	10	N.A.	R	AD
A3/61/F	3	1.5	4	9	N.A.	No	AD
A4/80/M	2	7.5	12	18	N.A.	R	AD
A5/65/M	3	3	16	9	N.A.	Mem and Ris	AD
A6/79/F	2	3.5	8	14	N.A.	R and T	AD
A7/72/F	3	9	0	3	N.A.	R, Ris, B and Cl	AD
A8/68/M	2	4	4	11	N.A.	D	AD
A9/83/F	1	5	5	22	N.A.	R	AD
A10/68/M	2	3	2	14	N.A.	R, Ris and Cl	AD
A11/77/F	2	3.5	4	11	N.A.	R and Mem	AD
A12/71/M	2	1.5	2	16	N.A.	No	AD
A13/82/F	2	1.8	0	17	N.A.	D	AD
A14/59/F	1	7	16	23	N.A.	R, Mem and Ris	AD
A15/80/F	2	3	0	15	N.A.	D and Ris	AD
A16/75/M	1	3	1	23	N.A.	Mem and Cl	AD
A17/61/F	2	1.2	4	13	N.A.	Ris	AD
A18/70/M	3	2	4	3	N.A.	R, Mem, E and Ris	AD
A19/78/F	1	1.5	3	16	N.A.	Mem and R	AD
A20/58/M	3	3.5	8	3	N.A.	D and Mir	AD
A21/85/F	2	1.5	4	13	N.A.	D, Ris and Cl	AD
D1/63/F	0	N.A.	4	26	17	Fl and Cl	Depression
D2/74/F	0.5	N.A.	4	25	12	Desv	Depression
D3/65/F	0.5	N.A.	1	25	19	P	Depression
D4/77/F	0	N.A.	8	26	14	Bus and Cl	Depression
D5/63/F	0.5	N.A.	0	24	14	S	Depression
D6/74/F	0.5	N.A.	4	24	14	Fl	Depression
D7/64/F	0.5	N.A.	1	26	15	V and Cl	Depression
D8/73/F	0.5	N.A.	0	19	16	No	Depression
D9/75/F	0.5	N.A.	2	25	16	C	Depression
H1/71/M	0	2	8	27	N.A.	N.A.	Hydrocephalus
H2/81/F	0	1	10	28	N.A.	N.A.	Hydrocephalus
H3/68/F	0	3	12	29	N.A.	N.A.	Hydrocephalus
H4/78/M	0	2	12	25	N.A.	N.A.	Hydrocephalus
H5/74/F	0	4	2	29	N.A.	N.A.	Hydrocephalus
H6/66/M	0	3	0	29	N.A.	N.A.	Hydrocephalus
H7/66/M	0	0.5	0	28	N.A.	N.A.	Hydrocephalus
H8/80/F	0	1	8	25	N.A.	N.A.	Hydrocephalus
H9/87/M	0	2	16	25	N.A.	N.A.	Hydrocephalus

CDR, Clinical Dementia Rating; MMSE, Mini Mental State Examination; AD, Alzheimer's Disease; F, Female; M, Male; N.A., Not Applicable; C, Citalopram; R, Rivastigmine; Mem, Memantine, Ris, Risperidone; T, Trazodone; B, Biperiden; Cl, Clonazepam; D, Donepezil; E, Escitalopram; Mir, Mirtazapine; Fl, Fluoxetine; Desv, Desvenlafaxine; P, Paroxetine, Bus, Buspirone; S, Sertraline; V, Venlafaxine.

Table S2 – Medication use does not affect glutamate and glutamine levels in probable AD patients.

AD	Glutamate (μmol/l)			Glutamine (μmol/l)		
	No users	Users	t-test (p value)	No users	Users	t-test (p value)
Rivastigmine	17.5 (2.28) (N=11)	17.1 (1.71) (N=10)	0.42 (0.68)	559.6 (133.4) (N=11)	506.0 (162.7) (N=10)	0.83 (0.42)
Memantine	16.9 (2.05) (N=15)	18.2 (1.64) (N=6)	-1.37 (0.19)	536.5 (126.0) (N=15)	527.9 (204.2) (N=6)	0.12 (0.91)
Risperidone	17.1 (2.19) (N=13)	17.5 (1.73) (N=8)	-0.42 (0.68)	546.4 (167.1) (N=13)	513.9 (113.9) (N=8)	0.48 (0.63)
Clonazepam	17.4 (2.15) (N=17)	16.8 (11.9) (N=4)	0.47 (0.64)	518.2 (147.7) (N=17)	601.6 (141.2) (N=4)	-1.02 (0.32)
Donepezil	17.3 (2.30) (N=16)	17.3 (0.21) (N=5)	-0.003 (0.99)	517.6 (158.7) (N=16)	586.8 (93.9) (N=5)	-0.92 (0.37)

Values are presented as means (standard deviation). Statistical significance is given by Unpaired t-test.

AD, Alzheimer's Disease.
